# Interdialytic blood pressure is independent of weight gain and correlated with serum angiotensin II levels in adults

**DOI:** 10.14814/phy2.70903

**Published:** 2026-05-10

**Authors:** Adolfo Diaz, Brenda Delgado, Eduardo R. Argaiz, Olynka Vega, Ricardo Correa‐Rotter, Bernardo Rodriguez‐Iturbe

**Affiliations:** ^1^ Department of Nephrology and Mineral Metabolism Instituto Nacional de Ciencias Médicas y Nutrición Salvador Zubirán Mexico City Mexico; ^2^ Instituto Tecnológico de Monterrey, Escuela de Medicina y Ciencias de la Salud Mexico City Mexico

**Keywords:** angiotensin, cardiac output, chronic kidney disease, hemodialysis, interdialytic weight

## Abstract

Chronic volume overload is a major risk factor of hypertension and mortality in chronic dialysis patients. However, the mechanisms responsible for hypertension are unclear. We studied 32 patients (age 23–68 years, 19 females) in chronic hemodiafiltration classified by their interdialytic ambulatory blood pressure (idABP, mmHg) in three groups: Group A: <130/80 (7 normotensives and 8 patients with controlled hypertension), Group B: 130–139/80–89 (insufficiently controlled hypertension, *n* = 7), and Group C: ≥140/90 (uncontrolled hypertension, *n* = 10). Studies included interdialytic weight gain (IDWG), idABP, predialysis blood pressure changes (Δ BP), cardiac output (CO), systemic vascular resistance (SVR), and serum concentrations of angiotensin II, norepinephrine, and copeptin. SVR and CO relationship and range were similar in all patient groups, but SVR was >1500 dynes/s/cm^−5^ in two‐thirds of the patients. Angiotensin II (pg/mL) was higher (*p* = 0.016) in group C (231.5 ± SD 127.8) than in group A patients (105.3 ± 46.5) and was positively correlated with CO (*p* = 0.002) and with idABP (*p* = 0.027). There were no significant differences in IDWG among the patient groups. idABP, Δ BP, CO, and SVR were unrelated to IDWG. Males had a higher (*p* = 0.045) systolic idABP than females. Results suggest that angiotensin II drives idABP independently of IDWG.

## 1 INTRODUCTION

Chronic kidney disease is the tenth leading cause of mortality worldwide and more than 3 million patients are currently receiving hemodialysis treatment several times weekly as renal replacement therapy (Flythe & Watnick, 2024; Romagnani et al., 2025). Weight monitoring is essential in dialysis patients because chronic volume overload is a risk factor of hypertension, cardiovascular complications, and mortality in patients in chronic dialysis (Agarwal et al., 2009; Inrig et al., 2007).

A consensus publication (Hecking et al., 2013) addressed the distinction between volume overload (defined as >15% above the individualized “dry” weight estimated by the nephrologist) and interdialytic weight gain (the oscillations in weight superimposed on the estimated dry weight that take place in the interval between dialysis). The relation of chronic volume overload with hypertension has been most notably shown in studies by Inrig et al. (2007) that found that a 1% increase in dry weight was associated with a 1 mmHg increase in predialysis systolic blood pressure and in the placebo‐controlled DRIP (Dry‐weight Reduction in Hypertensive Hemodialysis Patients) trial (Agarwal et al., 2009) that established that the loss of 1 kg of weight in 4–8 weeks obtained by progressive increase of ultrafiltration resulted in a mean reduction of 6.6 mmHg in systolic blood pressure and 3.1 mmHg in diastolic blood pressure.

In contrast, the interdialytic weight gain (IDWG) has unclear relations with interdialytic blood pressure (Kooman et al., 1992; Luik et al., 1994, 1997). IDWG is essentially the result of water and sodium retention, but it is not unusual to find interdialytic hypertension in patients that maintain their estimated dry weight, as well as normal blood pressure in patients with significant IDWG (Gudiño‐Bravo et al., 2025). Inconsistencies have been attributed to comorbidities and to the unreliability of blood pressure determinations in the dialysis unit that differ significantly from the interdialytic ambulatory blood pressure (idABP), that is the reference gold standard (Agarwal et al., 2006). In addition, pathophysiologic responses that modulate blood pressure and are known to be overactive in end‐stage kidney disease (Converse Jr et al., 1992; Hausberg et al., 2002; Kornerup et al., 1984; Neumann et al., 2004; Weidmann et al., 1971) likely participate in the regulation of interdialytic blood pressure, and their role has been largely unexplored.

The present study examined the relations of IDWG with hemodynamic blood pressure determinants (cardiac output and systemic vascular resistance), circulating levels of angiotensin II, norepinephrine, and antidiuretic hormone (ADH), and with idABP and predialysis blood pressure changes (Δ BP) in patients in a chronic hemodiafiltration program (HDF).

## 2 METHODS

### 2.1 General

This was an observational, prospective, hypothesis‐generating study approved by the Instituto Nacional e Ciencias Médicas y Nutrición Salvador Zubirán (INCMNSZ) ethical and scientific committee (INMM‐4512‐23‐24‐1) and registered in clinicaltrials.gov (NCT06764277). All patients gave informed consent for the study. Since bioimpedance and ultrasound methods to determine dry weight offer no clear advantage over the clinical assessment of progressive weight reduction (K/DOQI Workgroup, 2005; Loutradis et al., 2021), dry weight in our patients was estimated by the medical staff, probing with ultrafiltration the lower blood pressure tolerated by the patient without postural or dialysis‐associated hypotension or cramps.

Earlier guideline recommendations (K/DOQI Workgroup, 2005) of blood pressure levels for dialysis patients (predialysis <140/90 mmHg and postdialysis <130/80 mmHg) were re‐evaluated in 2015 and not enough evidence was found to support specific blood pressure targets for this population (National Kidney Foundation, 2015). In the present study, 7 patients were not receiving anti‐hypertensive treatment and had idABP <120/70 mmHg. The rest of the patients received anti‐hypertensive drug therapy. We classified our patients in 3 groups by their idABP: Group A: idABP <130/80 mmHg (7 normotensive patients and 8 patients with controlled hypertension); Group B: idABP 130–139/80–89 mmHg (7 patients with insufficiently controlled hypertension) and Group C: patients with idABP ≥140/90 mmHg (10 patients with uncontrolled hypertension).

### 2.2 Patients

**FIGURE 1:**
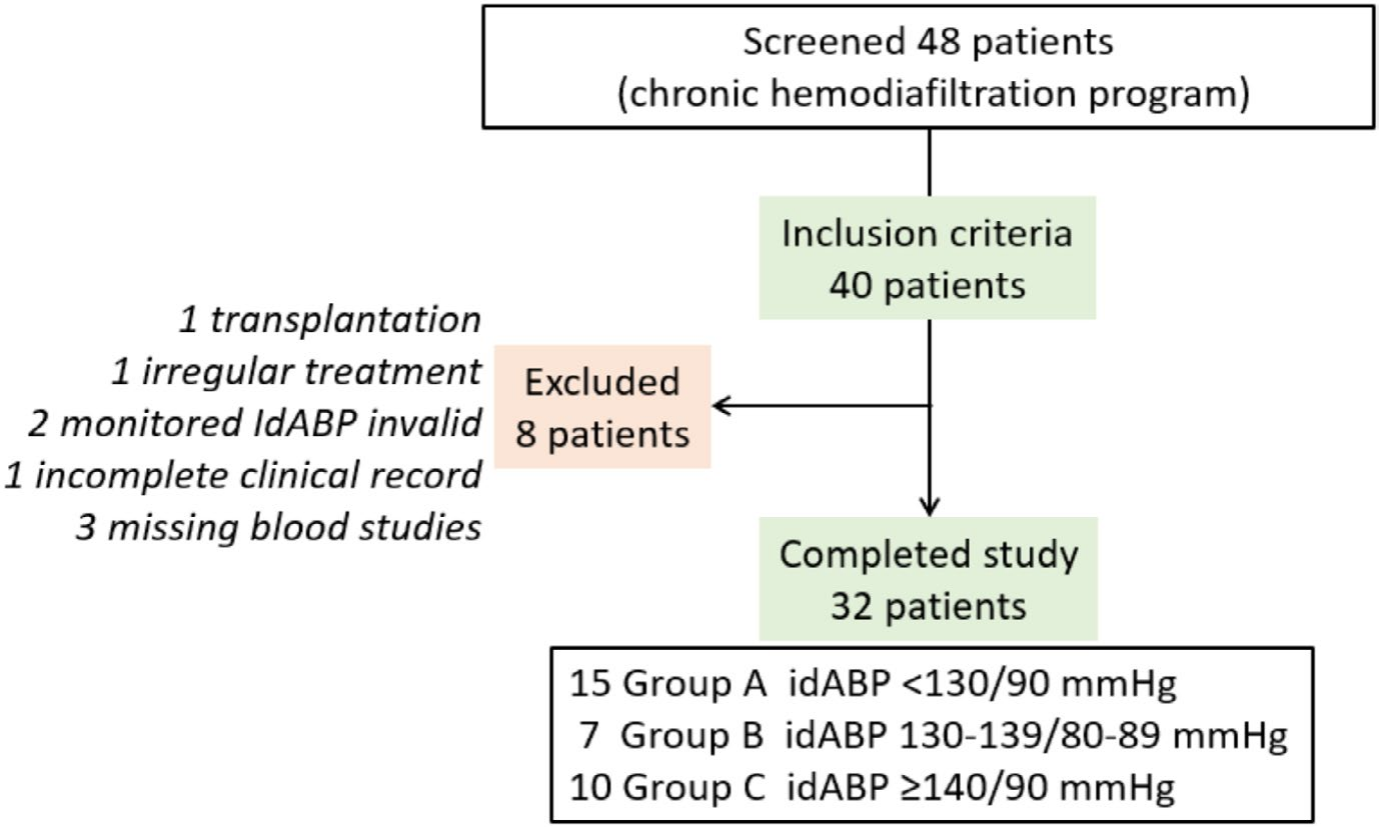
Flow diagram of study participants. Eight patients were excluded after initial inclusion for the following reasons: Transplantation in the interdialytic period (1 patient), irregular treatment that made it impossible to define stability of drug treatment (1 patient), and incomplete data in 6 patients (idABP monitoring not done or invalid in 2 patients; incomplete clinical record of 1 patient and lack of blood studies in 3 patients).

Studies were done in 32 patients (age 23–68 years, 19 females) of mixed race and ethnic background in the chronic HDF program of the INCMNSZ in Mexico City. All patients in the dialysis program are advised to keep a low sodium diet (<2.3 g/day), but no specific evaluation of sodium intake was done in the study. Patients were selected for the study with the following criteria: (1) 18 years of age or older; (2) more than 3 months in chronic hemodialysis; (3) stable clinical conditions with no evidence of infection and no changes in the prescription of medications for at least a month before the studies; (4) informed consent to participate in the study. Exclusion criteria were previous nephrectomy, administration of erythropoietin at the end of the HDF session in which the study was done, participation of the patient in another clinical trial that required clinical intervention, patients with pacemakers, incomplete data specified in the study protocol, and withdrawal of the consent to participate in the study. From a total of 48 patients in chronic HDF when the study was started, 40 fulfilled the inclusion criteria and eight were subsequently excluded (Figure 1).

### 2.3 HDF treatment and IDWG

Patients were treated with post‐dilutional HDF and a mean convective volume of 25 L. Treatments were administered for 4 h, 3 times weekly with CorDiax 5008S equipment (Fresenius Medical Care) and Fx100 dialyzers. The dialysis bath composition (Naturalyte, Fresenius Medical Care) was (mEq/L) Na 136–138, K 2, HCO_3_ 30–33, Ca 2.5. The studies were done in the short (2 days) interval between HDF sessions in 27 patients and in the long interval (3 days) in five patients. The ultrafiltration prescription was adjusted to restore the patient's estimated dry weight. In the days of the study, the patients used similar dressing and the same shoes, and their weight was determined by the nursing staff before and after HDF using a mechanical weight scale. IDWG was listed in kg and as the percent increment that the pre‐dialysis weight represented in relation to the weight at the end of the previous dialysis.

### 2.4 Dialysis blood pressure and ambulatory blood pressure monitoring

Systolic blood pressure (SBP) and diastolic blood pressure (DBP) were obtained by the dialysis equipment with the patient in the sitting position, both feet flat on the floor and the cuff in the upper arm at approximately heart level. Measurements were done before, every 30–60 min during, and at the end of the HDF session. Calibration and validation of the equipment are done by the provider once a year and locally (dialysis unit) whenever the registered blood pressure is unusually low or high.

Ambulatory blood pressure monitoring was done for 35.5 ± SD 1.1 (range 23–44) hours in the interdialytic period with either Space Labs Health Care, model 90217A, or Contec ABPM50. The blood pressure cuff was placed in the arm not used for venipuncture, 1–2 h after the HDF session and programmed for determinations every 30 min during the day and every hour during the night. There were 25.7 ± SD 5.6 (range 20–43) valid recordings per patient (Agarwal & Tu, 2018; Cepeda et al., 2023; Huang et al., 2021). Awake and sleeping periods were confirmed by the patient.

### 2.5 Cardiac output and systemic vascular resistance

Hemodynamic determinations were done in the interdialytic period, 2–3 h after the end of dialysis. Cardiac output (CO = systolic volume × heart rate) and central venous pressure (CVP) were determined by two experienced observers using Siemens Acuson P500 ultrasound equipment with a phased array transducer. Systolic volume was calculated with the velocity time integral of the flow of the left ventricle outflow tract of the aortic area. Systemic vascular resistance (SVR, dynes/s/cm^−5^) was calculated using MediCalc^R^ using the mean arterial pressure (MAP) and the equation: SVR = [(MAP−CVP) × 79.92]/CO.

### 2.6 Laboratory determinations

Serum concentrations of angiotensin II, norepinephrine, and copeptin (surrogate of ADH) were determined in blood samples drawn from the venous side of the vascular access at the end of the interdialytic period, before the initiation of the HDF. The differences between the pre and postdialysis concentrations were studied in 22 patients (Group A: *n* = 7, Group B: *n* = 6, and Group C: *n* = 9) with available postdialysis samples in addition to predialysis samples. Blood samples were allowed to coagulate at 2°C–4°C, and serum samples were stored at −80°C until the time of the assay. All determinations were done in the laboratory of the Department of Nephrology of the INCMNSZ. Angiotensin II was determined by ELISA (my BioSource Cat.# MBS703599); norepinephrine was determined by ELISA (ABCAM, Cat. # AB287789); copeptin was determined by ELISA (Bio‐techne/NovusBiological, Cat. # NBP2‐69822). Determinations were done following the instructions of the commercial supplier. Peripheral white cell counts were used to calculate the MNLR (monocytes + neutrophils/lymphocytes ratio) inflammation index that has been reported to be associated with adverse cardiovascular effects and hypertension (Chen et al., 2024; Li et al., 2025).

### 2.7 Statistical methods

Normal (Gaussian) distribution of the data was evaluated by Kolmogorov–Smirnov tests. Statistical differences between two groups were analyzed with *t*‐tests or, if data differed significantly from a normal distribution, with Mann–Whitney (unpaired data) or Wilcoxon (paired data) tests. Categorical data were analyzed with chi‐square (*X* ^*2*^) statistic. Comparisons between more than two groups were done with one‐way ANOVA and Tukey post‐tests or with Kruskal–Wallis and Dunn post‐test. Correlations were evaluated with Pearson or Spearman (rS) tests. Data outliers were identified by the ROUT method in data with normal distribution and excluded from analysis if Q 1% (false positive outliers <1%). Data are given as mean ± SD and 95% confidence interval, or as median and interquartile range if the data did not have a normal distribution. Two‐tail *p* values <0.05 were considered significant. GraphPad 10 was used for statistical calculations and graphs.

## 3 RESULTS

### 3.1 General data

Table 1 shows the general data of the patients in the study and the etiology of their chronic kidney disease. Dialysis vintage was widely variable and not significantly different in the patient groups. The number of patients in each group that were dialyzed via catheter reflects the limited number of arteriovenous fistulas that were adequate for HDF at the time of the studies. There were no significant differences in groups A, B, and C in serum hemoglobin levels (A = 9.3 ± 1.93 g/dL; B = 11.0 ± 3.44; C = 10.4 ± 1.58), serum sodium (A = 138 ± 3.87 mEq/L; B = 137 ± 3.17; C = 136 ± 2.85), serum potassium (A = 4.8 ± 0.77 mEq/L; B = 4.3 ± 1.32; C = 5.3 ± 1.58), serum chloride (A = 101 ± 4.3 mE/L; B = 98 ± 3.2; C = 99 ± 2.34), or serum bicarbonate (A = 24 ± 3.02 mEq/L; B = 24 ± 4.21; C = 23 ± 3.35).

**TABLE 1:** General data. Patients classified by interdialytic ambulatory blood pressure monitoring as normotensives and controlled hypertensives (Group A), insufficiently controlled hypertensives (Group B), and uncontrolled hypertensive patients (Group C).

Characteristics	Interdialytic ambulatory blood pressure			*p*
	<130/80 mmHg	130–139/80–89 mmHg	≥140/90 mmHg	
	(*n* = 15)	(*n* = 7)	(*n* = 10)	
Age years (median, range)	38 (23–68)	44 (33–72)	35 (26–77)	0.455*
Sex (F/M)	12/3	2/5	5/5	0.056*
Months in chronic dialysis (median, range)	27 (12–97)	46 (4–111)	51 (4–128)	0.365*
Vascular access: AVF/Catheter	5/10	2/6	1/9	0.410*
Body mass index	21.9 ± 3.36 (20.0–23.8)	21.5 ± 3.38 (17.9–25.0)	22.0 ± 3.80 (19.3–24.7)	0.956
Predialysis SBP (mmHg)	119.0 ± 15.72 (110.3–127.7)	144.3 ± 15.72 (129.7–158.8)	167.6 ± 25.51 (149.4–185.8)	<0.001
Predialysis DBP (mmHg)	66.4 ± 17.8 (59.9–72.9)	85.3 ± 8.9 (77.0–93.6)	93.1 ± 15.4 (82.1–104.1)	<0.001
idABP‐SBP (mmHg)	106.2 ± 9.83 (100.6–111.4)	132.4 ± 4.54 (128.2–136.6)	145.4 ± 12.7 (136.3–154.5)	<0.001
idABP‐DBP (mmHg)	65.4 ± 9.43 (60.2–70.6)	85.3 ± 2.29 (83.2–87.4)	88.8 ± 8.11 (83.0–94.6)	<0.001
Interdialytic weight gain (%)	2.90 ± 1.43 (2.11–3.69)	3.22 ± 1.68 (1.67–4.77)	2.98 ± 1.67 (1.79–4.17)	0.903
Dialysis Ultrafiltration (mL/h/kg)	7.48 ± 4.00 (5.26–9.69)	8.75 ± 3.56 (5.42–12.07)	9.36 ± 5.04 (5.76–12.96)	0.544
Etiology of CKD				
CKDu	8	2	2	0.484*
SLE	4	2	4	0.874*
Diabetic nephropathy	2	1	4	0.433*
ADPKD	1	1		
Transplant rejection (CKDu)		1		

*Note*: *p* Values calculated by ANOVA or *X* ^*2*^ statistics (*). Data are median and range or mean ± SD (95% CI).

Abbreviations: ADPKD, autosomal dominant polycystic kidney disease; AVF, arteriovenous fistula; CKD, chronic kidney disease; CKDu, CKD of undetermined cause; DBP, diastolic blood pressure; idABP, interdialytic ambulatory blood pressure; *n*, number of patients; SBP, systolic blood pressure; SLE, systemic lupus erythematosus; SVR, systemic vascular resistance.

Table 2 shows the hemodynamic characteristics, biomarkers and the medications that the patients were receiving for at least a month prior to the study. There were no significant differences in CO, SVR, norepinephrine, copeptin and MNLR in the patient groups. Angiotensin serum levels were higher (*p* = 0.016) in patients of group C than in patients of Group A. Normotensive patients of group A that did not receive antihypertensive treatment had lower serum angiotensin II (83.9 ± 22.2 pg/mL) than the normotensive patients that received treatment (123.6 ± 55.4 pg/mL), but the difference was not statistically significant (*p* = 0.129).

**TABLE 2:** Hemodynamic characteristics and biomarkers. Patients classified by interdialytic ambulatory blood pressure monitoring as normotensives and controlled hypertensives (Group A), insufficiently controlled hypertensives (Group B), and uncontrolled hypertensive patients (Group C).

Characteristics	Interdialytic ambulatory blood pressure (mmHg)			*p*
	Group A	Group B	Group C	
	<130/80	130–139/80–89	≥140/90	
	*N* = 15	*N* = 7	*N* = 10	
Cardiac index (L/min/m^2^)	2.62 ± 0.80 (2.13–3.10)	2.46 ± 0.48 (2.02–2.90)	2.68 ± 0.47 (2.35–3.01)	0.777
SVR (dynes/s/cm^−5^)	1513 ± 444 (1267–1759)	2045 ± 426 (1652–2439)	1751 ± 591 (1328–2174)	0.073
Angiotensin II (pg/mL)	105.3 ± 46.5 (n13)^¶^ (77.2–133.4)	158.3 ± 115.8 (51.2–265.4)	231.5 ± 127.9 (140.1–323)	0.016
Norepinephrine (pg/mL)	81.4 (n13)^Ψ^ (68.4–87.4)	82.4^Ψ^ (66.5–93.9)	82.6^Ψ^ (74.9–259.0)	0.677^Ψ^
Copeptin (pmol/L)	14.6 ± 3.79 (n12) (12.2–17.0)	10.3 ± 2.26 (n6) (7.92–12.65)	13.0. ± 4.67 (n9) (9.39–16.57)	0.11
MNLR	0.88 ± 0.51 (0.59–1.16)	0.73 ± 0.22 (0.49–0.95)	1.11 ± 0.40 (0.81–1.39)	0.224
Medications^a^				
ARB	1	5	4	
ARB + Sacubitril	5	0	2	
ACE inhibitors	1	0	0	
Ca channel blockers	0	6	6	
Beta receptor blockers	6	6	8	
Spironolactone	1	5	1	
Erythropoietin	10	5	5	
Immunosuppressors	5	2	2	

*Note*: Immunosuppressors include Cyclosporin A, mycophenolate mofetil, azathioprine. Data are mean ± SD (95% CI) or ^Ψ^median and interquartile range (data not compatible with normal distribution). *p* Values correspond to one‐way ANOVA or Kruskal–Wallis tests^Ψ^. ^¶^Two values removed as outliers determined by the ROUT *Q* = 1% (false positive values <1%). Serum angiotensin II values in groups A and C are significantly different (*p* = 0.012) as determined by Tukey post‐test after ANOVA.

Abbreviations: (*n*), number of studies when it was less than the number of patients; ACE, angiotensin converting enzyme; ARB, angiotensin II receptor 1 blockers; Sacubitril (neprilysin inhibitor); MNLR, monocyte neutrophil/lymphocyte ratio; *N*, number of patients; SVR, systemic vascular resistance.

^a^ Not listed other medications are Sevelamer, Cinacalcet, calcium carbonate, atorvastatin, hydroxychloroquine, pregabaline, clonazepam.

Drug therapy in the patient groups is also shown in Table 2. Only one patient was receiving an angiotensin converting enzyme inhibitor. Seventeen patients were receiving angiotensin receptor blockers alone (*n* = 10) or in combination with neprilysin inhibitors (*n* = 7). Calcium channel blockers and beta receptor blockers were prescribed to 12 and 20 patients, respectively.

### 3.2 Hemodynamic determinations and serum biomarkers

**FIGURE 2:**
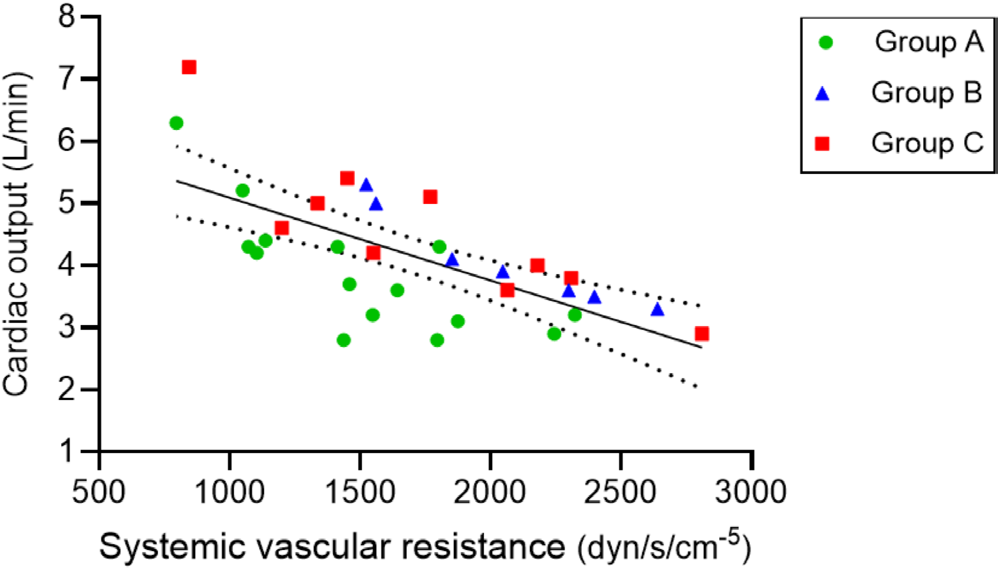
Hemodynamic characteristics. Patient groups are normotensives and controlled hypertensives (Group A), insufficiently controlled hypertensives (Group B), and uncontrolled hypertensives (Group C) (see text). Lines correspond to linear regression and 95% CI. Relationship between systemic vascular resistance and cardiac output. Slope −0.001 (95% CI −0.0018 to −0.0008), *r* = 0.673 (95% CI −0.83 to −0.423), *p* = <0.001.

Figure 2 shows the negative correlation between CO and SVR in the patients. The slope of the relationship was similar in the three patient groups, and the SVR was higher than 1500 dynes/s/cm^−5^ in 63% of the patients. The range of SVR and CO values is similar in all patient groups. To be noted, at any given range of SVR, the majority of group A patients have a lower CO than the rest.

**FIGURE 3:**
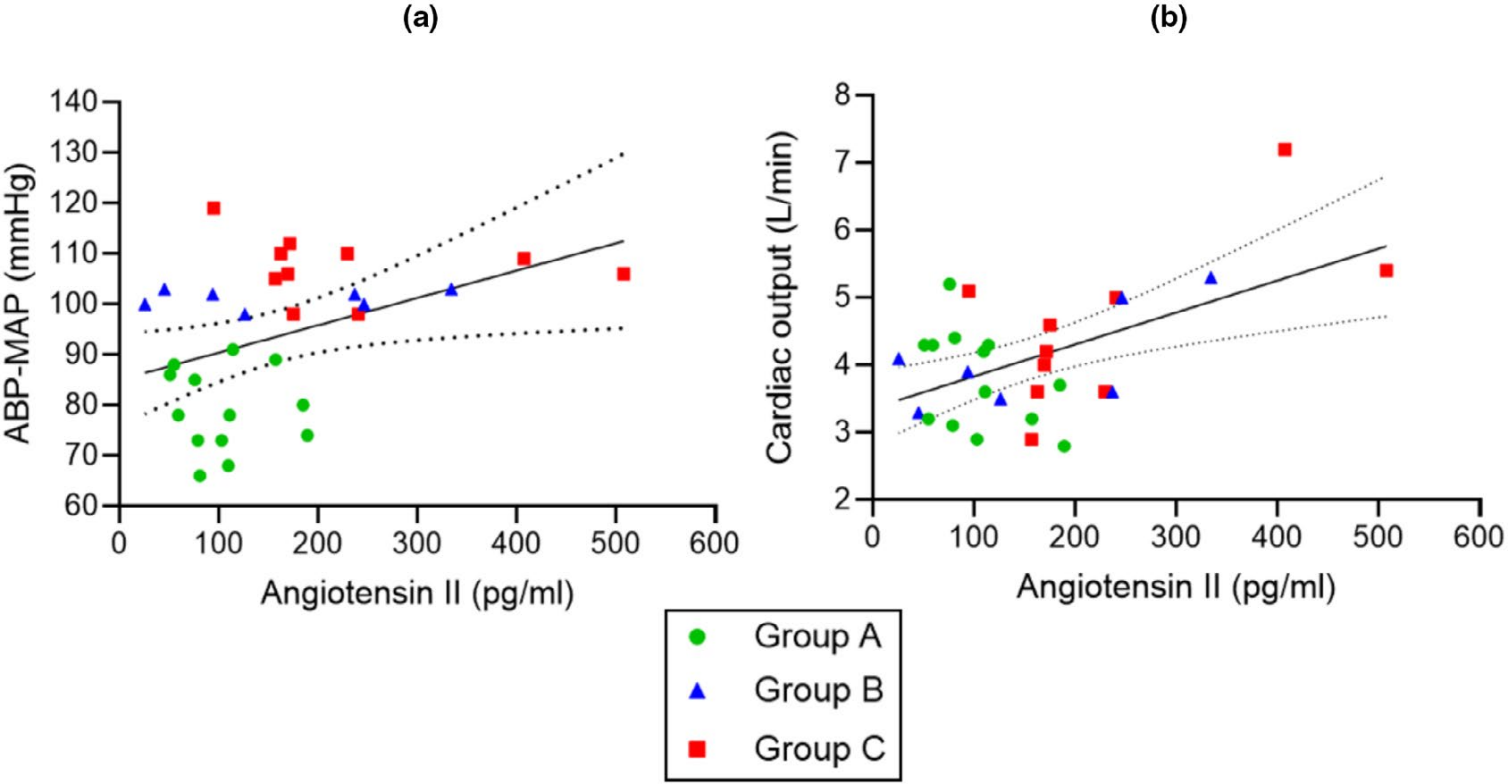
Angiotensin II serum levels, interdialytic ambulatory blood pressure and cardiac output. (a) Interdialytic (predialysis) angiotensin II levels correlation with interdialytic ambulatory mean arterial pressure (idABP‐MAP). Slope 0.054 (95% CI 0.0067–0.101), *r* = 0.404 (95% CI 0.051–0.667), *p* = 0.027. (b) Interdialytic (predialysis) Angiotensin II levels correlation with cardiac output. Slope = 0.0047 (95% CI 0.002–0.008); *r* = 0.540 (95% CI 0.22–0.75), *p* = 0.002. Patient groups are normotensives and controlled hypertensives (Group A), insufficiently controlled hypertensives (Group B), and uncontrolled hypertensives (Group C) (see text). Lines correspond to linear regression and 95%CI.

Interdialytic levels of angiotensin II had a correlation of borderline significance (*p* = 0.027) with the severity of hypertension (Figure 3a) and a robust correlation with CO (*p* = 0.002) (Figure 3b).

**FIGURE 4:**
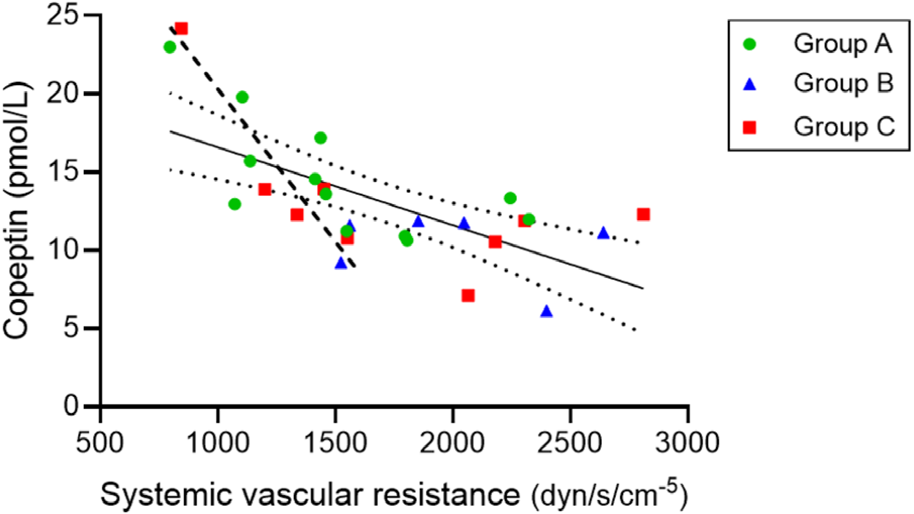
Copeptin levels and systemic vascular resistance. Negative correlation between systemic vascular resistance and interdialytic Copeptin levels: Slope −0.005 (95% CI −0.001 to −0.002), *r* = −0.659 (95% CI −0083 to −0037), *p* = 0.0002. Interrupted line corresponds to data of ≤1500 dynes/s/cm^−5^ of systemic vascular resistance.

Unexpectedly, there was a negative correlation between interdialytic Copeptin levels and SVR that is steeper at levels of SVR below 1500 dynes/s/cm^−5^ (interrupted line in Figure 4).

Interdialytic changes in serum biomarker levels were studied in 22 patients in association with blood pressure and weight changes (Table 3). Copeptin levels were significantly reduced in the interdialytic period (*p* < 0.001). Post and predialysis levels of Angiotensin II and Norepinephrine were unchanged and did not show the reduction expected with a weight gain of about 2 kg (presumably due to water retention) (Table 3).

**TABLE 3:** Interdialytic changes.

	Post dialysis	Predialysis	*p*
Copeptin (pmol/L)	26.7 ± 12.9 (20.9–32.4)	12.4 ± 3.6 (10.3–14.4)	<0.001
Angiotensin (pg/mL)	190.7 ± 192.5 (105.3–276)	188.3 ± 122.5 (134.0–242.6)	0.962
Norepinephrine (pg/mL)	81.4^Ψ^ (71.3–112.8)	84.0^Ψ^ (72.4–92.0)	0.925^Ψ^
Systolic blood pressure (mmHg)	132.3 ± 18.5 (124.1–140.5)	143.4 ± 29.0 (130.5–156.2)	0.024
Diastolic blood pressure (mmHg)	79.3 ± 14.4 (72.9–85.7)	82.2 ± 3.81 (74.2–90.1)	0.364
IDWG (Kg)	58.6 ± 15.9 (51.6–65.7)	60.5 ± 16.3 (53.3–67.8)	<0.001

*Note*: Determinations in 22 paired post and pre dialysis samples. Data for Copeptin and angiotensin are mean ± SD (95% CI). Data are mean ± SD, except ^Ψ^data for norepinephrine that do not have a normal distribution are median and 25%–75% IQR. *p* Values correspond to *t*‐tests or ^Ψ^Wilcoxon test.

Abbreviation: IDWG, interdiaytic weight gain.

### 3.3 The independence between IDWG and blood pressure

**FIGURE 5:**
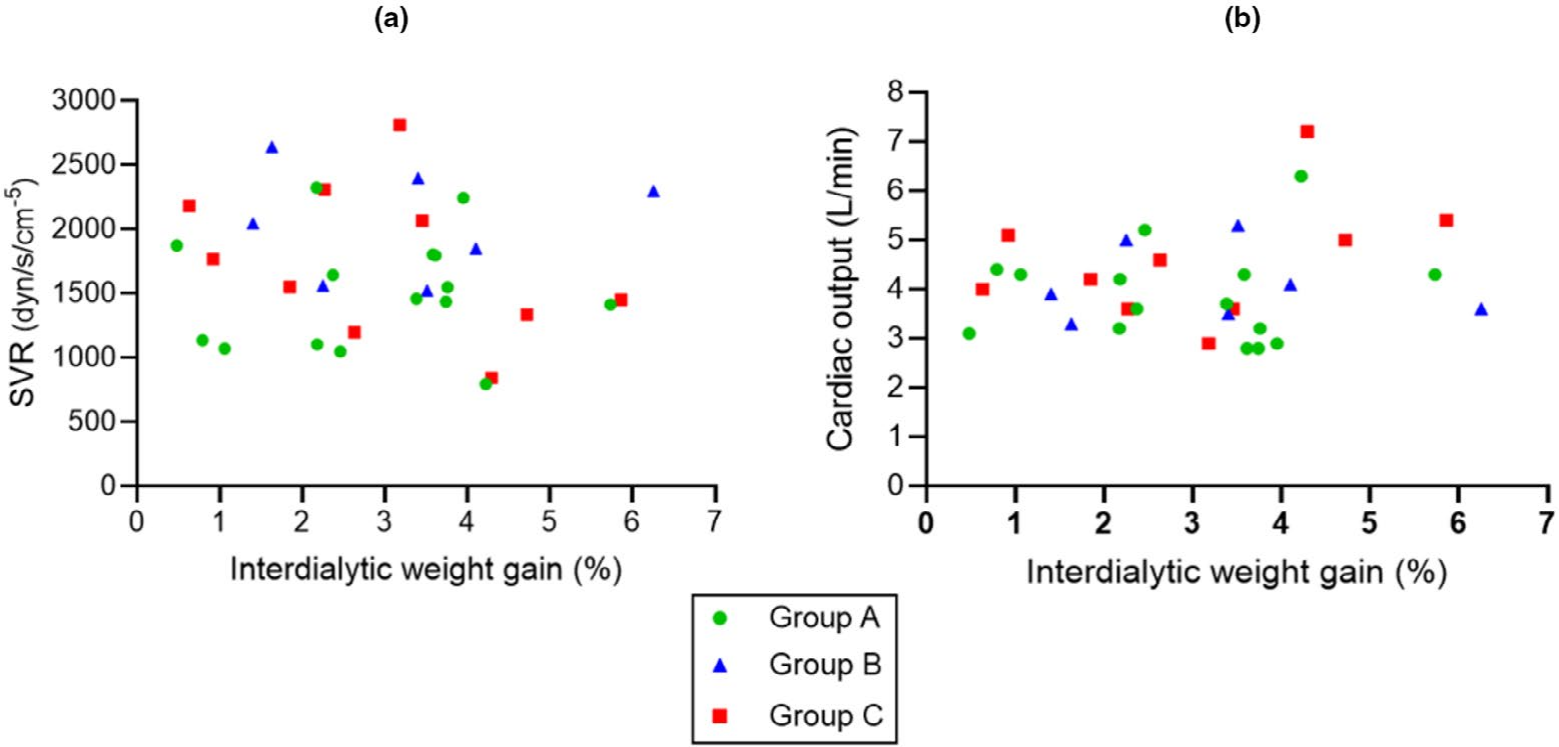
Interdialytic weight gain, systemic vascular resistance (SVR), and cardiac output. Group A (normotensive patients), Group B (insufficiently controlled hypertensive patients), Group C (uncontrolled hypertensives) (see text). (a) All patients: *p* = 0.631; Group A: *p* = 0.860; Group B: *p* = 0.987; Group C: *p* = 0.268. (b) All patients: *p* = 0.365; Group A: *p* = 0.836; Group B: *p* = 0.836; Group C: *p* = 0.261.

**FIGURE 6:**
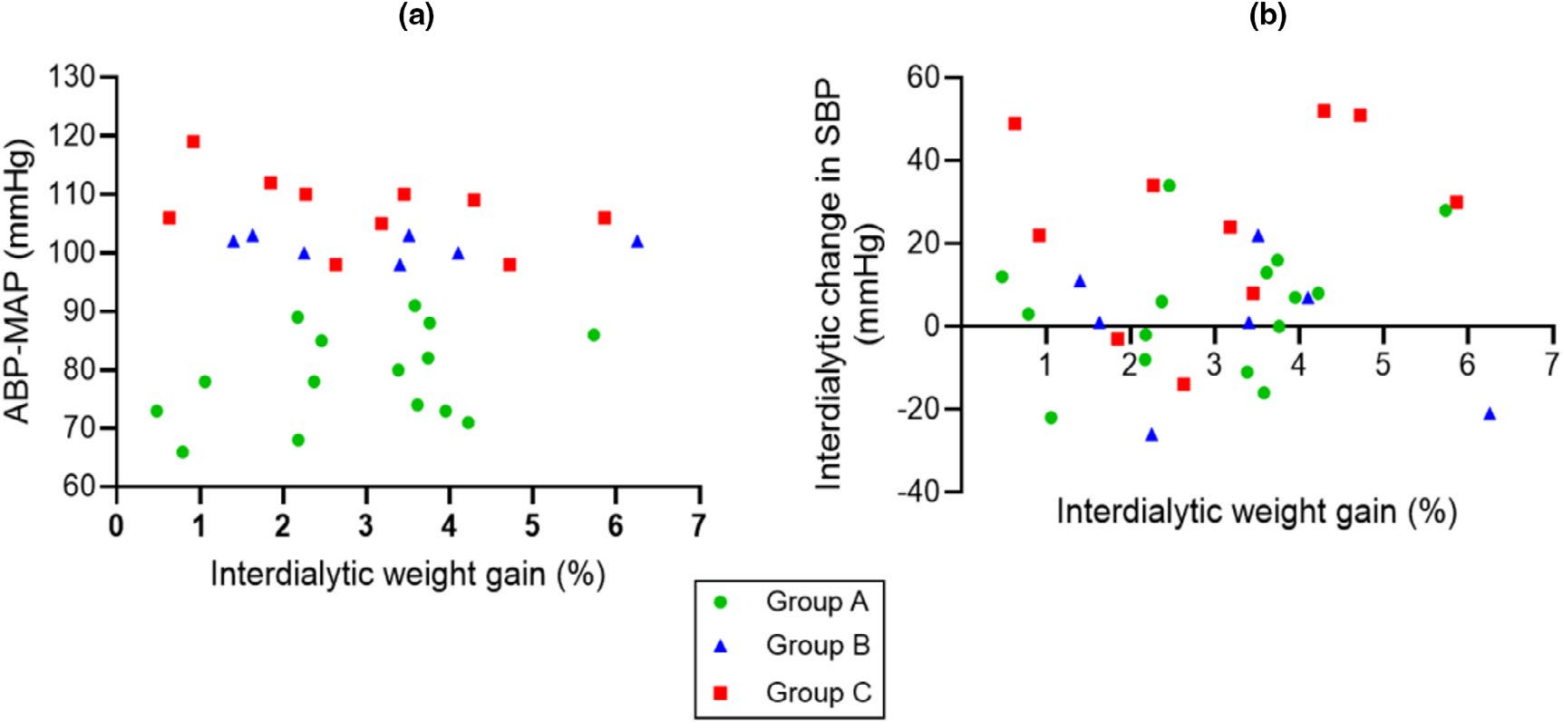
Independence of interdialytic weight gain and blood pressure. (a) Interdialytic weight gain and ambulatory mean blood pressure (idABP‐MAP) (*p* = 0.727). (b) Interdialytic weight gain and interdialytic change in SBP pressure (Predialysis – Postdialysis) (*p* = 0.520). Patient groups are normotensives and controlled hypertensives (Group A), insufficiently controlled hypertensives (Group B) and uncontrolled hypertensives (Group C) (see text).

Figure 5a,b show the lack of relation between IDWG and SVR and CO. Figure 6a,b show the independence of idABP and Δ BP from IDWG. As shown in Figure 6b, nine patients had an interdialytic reduction (−1 to −26 mmHg) in blood pressure, despite a 1% to 6.3% increase in interdialytic weight and, in contrast, an increase in SBP blood pressure of up to 50 mmHg was found in some patients who gained <1% of weight between dialysis.

**FIGURE 7:**
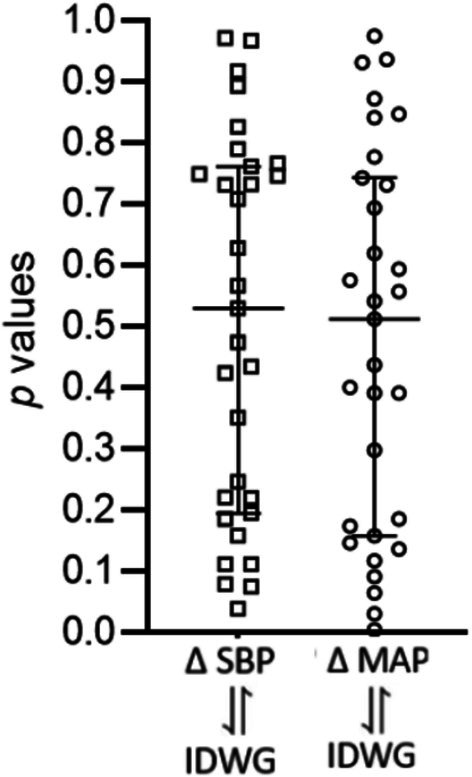
Correlations between changes in blood pressure and changes in weight in 7 additional hemodialysis in each patient of the study. Each sign corresponds to the *p* value of the correlation between change in systolic blood pressure (∆SBP) and mean blood pressure (∆MAP) with the interdialytic weight gain (IDWG) in 7 studies of 1 patient. Bars indicate median and IQR.

We then evaluated the relation between changes in interdialytic weight and changes in systolic and mean pressure in seven additional HDF sessions of each patient in the study. As shown in Figure 7, associations had an average probability of resulting from chance alone (50%).

### 3.4 Sexual differences

Because there are sexual differences in the pathophysiology of blood pressure, we compared the characteristics of the female and male participants of our study (Table 4). Male patients had a higher idABP‐SBP than females (*p* = 0.045). No other significant differences were found between female and male patients.

**TABLE 4:** Sexual characteristics.

	Females (*N* = 19)	Males (*N* = 13)	*p*
Age, years (median, range)	33 (23–68)	42 (26–72)	0.537
Cardiac output (L/min)	4.1 ± 1.01 (3.6–4.6)	4.2 ± 1.11 (3.6–4.9)	0.709
Systemic vascular resistance (dynes/s/cm^−5^)	1678 ± 523 (1426–1930)	1742 ± 533 (1420–2064)	0.916
idABP‐SBP (mmHg)	118.3 ± 16.9 (110.1–126.4)	132.9 ± 22.6 (119.2–146.5)	0.045
idABP‐DBP (mmHg)	74.9 ± 14.3 (68.0–81.9)	80.2 ± 12.5 (72.7–87.8)	0.290
IDWG (%)	3.09 ± 1.53 (2.35–3.83)	2.86 ± 1.54 (1.93–3.78)	0.965
Angiotensin II (pg/mL)	153.6 ± 119.2 (n17)^¶^ (92.3–214.8)	168.5 ± 96.9 (109.9–227.1)	0.716
Norepinephrine (pg/mL)	83.6^Ψ^ (n17) (70.5–89.1)	77.5^Ψ^ (70.3–129.7)	0.805^Ψ^
Copeptin (pmol/L)	13.0 ± 3.6 (n16) (11.1–14.9)	13.1 ± 4.91 (n11) (9.8–185.3)	0.950
MNLR index	0.93 ± 0.52 (0.68–1.18)	0.91 ± 0.32 (0.71–1.11)	0.874
Ultrafiltration (mL/kg/h)	9.28 ± 4.52 (7.10–11.46)	6.97 ± 3.45 (4.88–9.05)	0.129
CKD etiology			
Lupus nephropathy	8	2	
Diabetic nephropathy	2	4	
CKDu (etiology unknown)	7	5	
ADPKD	1	1	
Other	1	1	

*Note*: Data are mean ± SD (95% CI) analyzed by unpaired *t*‐tests or ^Ψ^median and interquartile range analyzed by Mann–Whitney tests (data not compatible with normal distribution). ^¶^Two values removed as outliers determined by the ROUT *Q* < 1%.

Abbreviations: (*n*), number of studies when it was less than the number of patients; ADPKD, autosomal dominant polycystic kidney disease; CKDu, chronic kidney disease of unknown etiology; DBP, diastolic blood pressure; idABP, interdialytic ambulatory blood pressure; *N*, number of patients; NMLR, monocyte neutrophil ratio; SBP, systolic blood pressure.

## 4 DISCUSSION

The pathophysiology of hypertension in patients in chronic kidney disease is multifactorial and the prognostic relevance of chronic volume overload is well recognized (Bansal et al., 2023) and the reduction in blood pressure resulting from 4 to 8 weeks of downward probing the dry weight with ultrafiltration has been conclusively demonstrated (Agarwal et al., 2009). However, the interdialytic period, characterized by a transition from postdialysis volume contraction to predialysis expansion, occurring 3 times weekly in patients in chronic dialysis, has received relatively little attention.

We did the evaluation of CO and SVR 2–3 h after the end of the HDF session because at that time the hemodynamic instability induced by dialysis ultrafiltration had subsided and the mean values of CO and SVR are within 10% of the values found at the end of the interdialytic period (Doenyas‐Barak et al., 2019; Strangfeld et al., 1973). The Copeptin levels are reduced in the interdialytic period, as expected by the water retention, but angiotensin II and norepinephrine concentration, which are the most important physiological modulators of CO and SVR in the interval between dialysis, did not have consistent postdialysis‐to‐predialysis changes (Table 3). In these patients, the systolic blood pressure increased during the interdialytic period without changes in serum angiotensin II. To be noted, the appropriate physiological reduction in angiotensin II levels in response to water retention of about 2 liters (IDWG of 2 kg in Table 3 are mostly due to sodium and water retention) was not observed. Therefore, the inappropriately stable angiotensin II levels despite volume expansion likely contributed to the increased blood pressure.

Two‐thirds of the patients had an increased SVR (>1500 dynes/s/cm^−5^). Both kidneys were present in our patients and they likely played a role in the high SVR since previous studies (Kim et al., 1972) have shown that nephrectomy importantly reduces the SVR. In addition, the combined activity of the renin‐angiotensin and sympathetic systems and ADH system contributes to the high incidence of increased SVR.

A surprising finding was the negative correlation between interdialytic copeptin serum level and SVR (Figure 4), which is opposite to the expected because increased ADH activity, stimulated by osmotic and non‐osmotic stimuli, acting on V1 receptors induces vasoconstriction (Gao et al., 2025). The possibility of osmotic stimulation of ADH in the interdialytic period is unlikely since there was an important reduction of predialysis copeptin levels (Table 3) associated with IDWG. One of the non‐osmotic stimuli of ADH is vasodilatation, that elicits a protective response that is known to occur in sepsis when the fall of SVR threatens blood pressure (Gomes et al., 2021). It is conceivable that the negative interdialytic correlation between copeptin levels and SVR represents an ADH response triggered by and directed to correct a reduction in SVR. This possibility is in line with the steeper increase in copeptin levels when values of SVR are lower than 1500 dynes/s/cm^−5^ (interrupted line in Figure 4). Obviously, a protective response, if such were the case, appears to start at higher than normal SVR levels in the interdialytic period and, therefore, requires clarification in subsequent investigations.

Our study found that serum angiotensin II levels were higher in patients with idABP ≥140/90 mmHg (Table 2). As noted earlier, the slope and range of the relation between SVR and CO are similar in all patient groups. However, at any range of SVR, the majority of patients in group A have lower CO than the rest (Figure 2), suggesting that in the interval between dialysis, despite increased SVR, hypertension is predominantly driven by a higher CO. The angiotensin‐driven increment in CO is well‐recognized (DeMello & Danser, 2000) and the angiotensin II levels that are borderline correlated with the idABP (Figure 3a) are also correlated with CO (Figure 3b). The overactivity of the renin‐angiotensin system in patients with chronic kidney disease has been established (Kornerup et al., 1984; Weidmann et al., 1971) and treatment with angiotensin converting enzyme inhibitors and angiotensin receptor blockers has been associated with reduced mortality (Lee et al., 2018), but the correlation between angiotensin serum levels and idABP and CO in the interdialytic period has not been, to our knowledge, reported previously.

There are well‐characterized sexual differences in blood pressure regulation (Drury et al., 2024). We found that males had a higher idABP‐SBP (Table 4). No other differences were found between female and male patients. In the general population, the lower premenopausal blood pressure levels are a result, in large measure, of estrogen interactions with the renin‐angiotensin system that activate the vasodilation pathway Ang(1‐7)‐MasR‐AT2R, reduce ACE expression and counteract Ang II‐AT1R‐activity (Medina et al., 2020). The microvascular vasodilatation activity of the Ang(1–7) pathway is lost in postmenopausal females (Schwartz et al., 2023). Our study was not designed for evaluating sexual differences and we did not study estrogen levels in our female patients. A large study of dialysis patients found that only 44% of the patients had postmenopausal serum estrogen E1 levels (<15 pg/mL) and only 30% had low total serum E2 levels (<5 pg/mL) (Kramer et al., 2003). Therefore, an important number of our female participants likely had sufficient estrogen levels to exert a modulatory role on the angiotensin II‐AT1R vasoconstriction and thereby explain the higher blood pressure in male patients.

Increased sympathetic activity has been reported in CKD before (Converse Jr et al., 1992; Hausberg et al., 2002; Neumann et al., 2004) and could be a potential cause of hypertension. A recent investigation suggested that the lack of an inverse relation between sympathetic activity and expanded extracellular volume was a manifestation of chronic kidney disease of relevance in hypertension (Jeong et al., 2026). Along the same lines, there was no reduction in norepinephrine levels associated with a water retention of about 2 L in the interdialytic period (Table 3) but definite conclusions require evaluation of sympathetic activity that was not examined in our studies.

The MNLR index has also been associated with increased risk of adverse outcomes and hypertension (Chen et al., 2024; Li et al., 2025) but in our patients the MNLR was low and there were no significant differences in our patient groups (Table 2) nor between sexes (Table 4). MNLR is not a surrogate of inflammatory cytokines and therefore, the potential role played by inflammation (Rodriguez‐Iturbe et al., 2017) in interdialytic hypertension needs to be examined in subsequent studies.

The independence of volume expansion and blood pressure in dialysis patients has been noted before. Luik et al. (1997) studied dialysis patients with imposed changes of −1 kg to +3 kg and found that blood pressures were similar in both periods. Shantier et al. (2019) found that the greater IDWG that occurs in the long vs. the short interval between dialysis had a minor, if any, effect on blood pressure. Wabel et al. (2008) in a study of 500 patients from eight dialysis units found that volume overload evaluated by bioimpedance was associated with hypertension in only 15% of the patients.

In summary, our study showed that idABP, Δ BP, SVR, and CO are unrelated to IDWG. Two thirds of the patients have increased SVR independently of the blood pressure control. The range and slope of CO and SVR were similar in dialysis patients with normal, insufficiently controlled, and uncontrolled hypertension. However, at all ranges of SVR, most normotensive patients had lower CO than the rest, and serum levels of angiotensin II were correlated with CO and with idABP.

Water and sodium retention are both hallmarks of end‐stage kidney disease relevant in the pathogenesis of hypertension (Mayeda & Bansal, 2026). Our data should not be interpreted as minimizing the beneficial effects of maintaining dry weight or suggesting preferential use of some drug therapies. Our results posit that emphasis in the blockade of the renin‐angiotensin system could accomplish more successfully blood pressure control, independently of IDWG.

There are limitations in our study. The patients were receiving several medications and while there were no changes in at least a month before the studies, drug‐related interactions are expected. One patient was receiving an ACE inhibitor that could have reduced the levels of serum angiotensin II and seven patients (5 patients of group A and 2 patients of group C) were taking a combination of neprilysin inhibitor and Losartan, that could result in neprilysin‐induced increased levels of serum angiotensin II. Nevertheless, removing these patients from the calculations did not change the results (difference between serum angiotensin II in group A and group C, *p* = 0.020; correlation between angiotensin II serum levels and cardiac output *r* = 0.589, 95% CI 0.233–0.806, *p* = 0.031). Another limitation is that our study was done in a single interdialytic period and variability of the data in serial determinations was not explored, however, the independence of weight changes and blood pressure changes was also demonstrated in seven additional studies in each patient (Figure 7). Finally, classification of patients with normal, insufficiently controlled and uncontrolled interdialytic ambulatory hypertension is admittedly arbitrary; however, correlation studies allowed evaluations of the blood pressure continuum, in addition to blood pressure categories. We acknowledge that our work was done in a limited number of patients of a single institution and the results should be considered hypothesis‐generating and require confirmation in multicentric investigations.

## AUTHOR CONTRIBUTIONS

**Adolfo Diaz:** Investigation; methodology. **Brenda Delgado:** Investigation; methodology. **Eduardo R. Argaiz:** Investigation; methodology; validation. **Olynka Vega:** Supervision. **Ricardo Correa‐Rotter:** Conceptualization; formal analysis. **Bernardo Rodriguez‐Iturbe:** Conceptualization; formal analysis; investigation; methodology; supervision; validation.

## FUNDING INFORMATION

Investigation research funds of the Department of Nephrology and Mineral Metabolism, INCMNSZ.

## CONFLICT OF INTEREST STATEMENT

None of the authors have conflicts of interest related to this work.

## ETHICS STATEMENT

Studies were conducted with adherence to NIH ethic standards and approved by the institutional ethic committee.

## Data Availability

All data are included in the manuscript. Data of individual unidentified participants will be available from 2 months to 2 years after publication to researchers with a reasonable request to the corresponding author.
